# Diel vertical migration rates of the dinoflagellate species *Margalefidinium polykrikoides* in a lower Chesapeake Bay tributary

**DOI:** 10.3389/fmicb.2024.1378552

**Published:** 2024-11-13

**Authors:** Sophie Clayton, Jacqueline B. Chrabot, Michael Echevarria, Leah Gibala-Smith, Kathryn Mogatas, Peter Bernhardt, Margaret R. Mulholland

**Affiliations:** ^1^National Oceanography Centre, Southampton, United Kingdom; ^2^Department of Ocean and Earth Sciences, Old Dominion University, Norfolk, VA, United States

**Keywords:** diel vertical migration, harmful algal blooms, dinoflagellates, swimming speed, mixotrophic phytoplankton

## Abstract

*Margalefidinium polykrikoides* is a mixotrophic dinoflagellate harmful algal bloom (HAB) species that blooms annually in the lower Chesapeake Bay. *M. polykrikoides* undertakes a diel vertical migration (DVM) which may give it a competitive advantage over purely phototrophic organisms who cannot access deeper nutrient pools and allow it to form large toxic blooms. Laboratory-based estimates of *M. polykrikoides’* DVM rates suggest that it is one of the fastest known dinoflagellate swimmers and understanding this behavior is likely important for modeling and predicting *M. polykrikoides* blooms. However, to date, no field-derived estimates of *M. polykrikoides’* DVM rates have been made in the Chesapeake Bay. In this study, we conducted four targeted field experiments to investigate the DVM of *M. polykrikoides* in the Lafayette River, a sub-tributary of the Chesapeake Bay. Vertical profiles of chlorophyll *a* fluorescence collected at least every 2 h over diel periods were used to track the DVM of *M. polykrikoides* during blooms. The maximum observed DVM rate for *M. polykrikoides* was 2.5 m h^−1^, with mean DVM rates around 1.3 m h^−1^ for both ascents and descents. As in studies from other regions, our results show that *M. polykrikoides’* ascent to/descent from the surface initiates before sunrise/sunset, suggesting phototaxis is not the primary trigger of their DVM. However, unlike in other studies where *M. polykrikoides* was observed to modulate its DVM to avoid excessively warm temperatures (≥30°C), we do not observe active thermotaxic avoidance, despite ambient temperatures exceeding their optimal threshold.

## 1 Introduction

Harmful algal blooms (HABs) are increasing in their frequency and duration throughout the Chesapeake Bay and globally (Hallegraeff, 2003; Mulholland et al., 2009, 2018). HAB events can result in the introduction of toxins into the water column, cause reductions in dissolved oxygen (DO) as they decay and impact pH levels, all leading to significant losses to aquaculture, recreational and commercial fisheries, and the tourism industry (Harding et al., 2009; Mulholland et al., 2009; Friedland et al., 2011; Reece et al., 2012). In the Chesapeake Bay, in particular, HABs are predicted to become larger and more frequent due to climate change (Najjar et al., 2010). HAB related impacts on the shellfish industry on the east coast of the United States, including the Chesapeake Bay, are likely to grow as this industry is projected to increase in economic value and geographic expanse over the next decade (Hudson, 2019). As a result, there is a strong need to better understand not only the environmental drivers of HABs but also the ecological and physiological capabilities of HAB organisms that allow them to outcompete other phytoplankton and form toxic blooms in the environment.

*Margalefidinium polykrikoides* (formerly *Cochlodinium polykrikoides*) is a mixotrophic dinoflagellate HAB species that has bloomed nearly annually in the lower Chesapeake Bay since at least 1986 (Marshall and Egerton, 2009; Mulholland et al., 2018). Blooms of *M. polykrikoides* can persist from weeks to months and extend from the York River to the coastal waters off Virginia Beach, VA, United States (Morse et al., 2013; Mulholland et al., 2018; Xiong et al., 2022). Due to their spatial heterogeneity and ephemeral nature, the magnitude, frequency, and duration of these blooms are likely under-represented by routine water quality monitoring programs that sample monthly at fixed stations (e.g., the Chesapeake Bay Monitoring Program), contributing to the difficulty in studying and understanding their dynamics.

Most dinoflagellate HAB species have complicated life cycles and employ a variety of behaviors and metabolisms that are thought to enhance their growth and competitive abilities (Kudela et al., 2008; Hansen, 2011). As mixotrophs, organisms that use both photoautotrophy and heterotrophy to acquire carbon, it has been hypothesized that by undertaking a diel vertical migration (DVM), *M. polykrikoides* can take advantage of high light levels near the surface during the day to support photosynthetic carbon acquisition while accessing inorganic and organic nutrient pools near the bottom at night, giving them a competitive advantage over strictly phototrophic organisms (Hansen, 2011). *M. polykrikoides’* growth rates are strongly controlled by temperature, and laboratory studies have shown that their specific growth rate drops off steeply at temperatures above their thermal optimum range of 21–26°C (Kim et al., 2004). Recent work has further suggested that *M. polykrikoides* may modulate their vertical migration rates in response to thermotaxic stimuli to adjust their position in the water column to avoid thermal stress (hot or cold), and thereby maintain a thermally optimal position in the water column for growth (Lim et al., 2022). In estuarine systems like the lower Chesapeake Bay where strong thermal stratification develops in the summer months when blooms are most likely to form, this combination of vertical migration and thermotaxis may provide an additional competitive advantage over other phytoplankton species that cannot regulate their thermal environment by migrating through the water column.

A lab-based study estimated *M. polykrikoides’* mean swimming speed at 3.82 m h^−1^ (Jeong et al., 1999), making them one of the fastest known swimmers among mixotrophic dinoflagellate species. Although field estimates of *M. polykrikoides* DVM rates, ranging from 0.47 to 4 m h^−1^, have been made in Korean coastal waters (Park et al., 2001; Kim et al., 2010; Lim et al., 2022), no such estimates have been made for the strain of *M. polykrikoides* found in the Chesapeake Bay. In this study, we derived *M. polykrikoides’* DVM rates from data collected during four separate 24-h experiments at a time series site in the Lafayette River, a sub-tributary of the Lower Chesapeake Bay. In the following sections, we describe our field site and sampling methods, our method for estimating *M. polykrikoides* DVM rates from sequential fluorescence profiles, we report estimates of *M. polykrikoides’* maximum and mean DVM rates and discuss them in the context of the environmental conditions in the water column.

## 2 Methods

### 2.1 Study site

**Figure 1:**
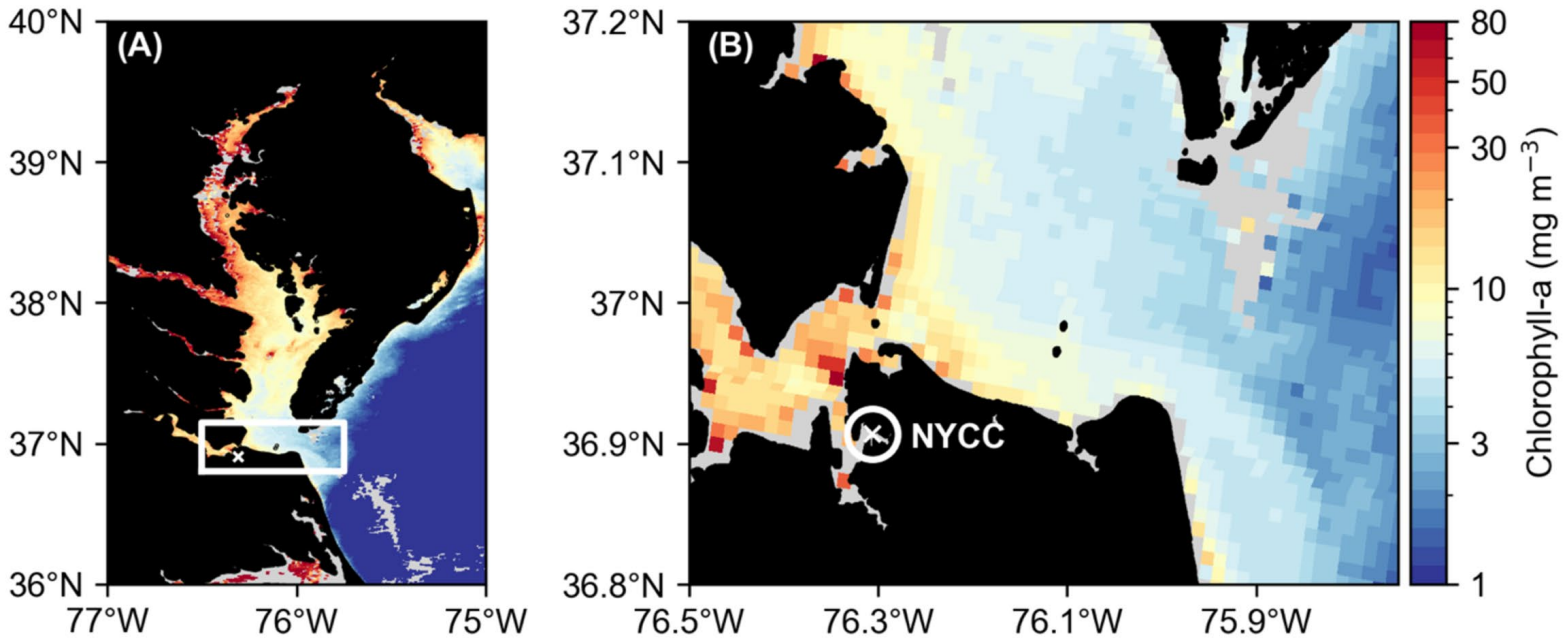
Map of the larger Chesapeake Bay region **(A)** indicating the lower Chesapeake Bay study region with a white rectangle and a white cross showing the location of the Norfolk Yacht and Country Club (NYCC) time series site, located on the Lafayette River, where the Diel Studies were conducted. A closer view of the study region **(B)** with a white circle indicating the Lafayette River and a white cross indicating the location of the NYCC site. In both panels, the background colormap shows the surface chlorophyll *a* concentration (mg m^−3^; MODIS Terra Level 2) on 22/8/2016, when DS2 was conducted. Note the logarithmic color scale.

The Lafayette River, a sub-tributary of the lower James River in the lower Chesapeake Bay, is known to be a hotspot for *M. polykrikoides* bloom initiation (Morse et al., 2013; Mulholland et al., 2018). A time series site in the Lafayette River at the Norfolk Yacht and Country Club (NYCC; Figure 1), has been used for weekly to sub-daily sampling each summer (June to September) since 2012, to target the onset, proliferation, and decline of summer HABs, particularly *M. polykrikoides*. The average water depth at NYCC is 6 m, and it has a 1–2 m tidal range. As part of the routine time series sampling, water column profile data are collected and water samples are collected for several analyses, including chlorophyll *a* (hereafter abbreviated to Chl-a) concentration and phytoplankton identification and cell enumeration. In addition to the routine sampling, four separate “Diel Studies,” designed to capture and quantify the vertical migration patterns and rates of *M. polykrikoides* were undertaken during the summers of 2016 and 2021 when *M. polykrikoides* was greater than 80% of the total phytoplankton biomass. The data from the routine sampling were used to track the temporal progression of *M. polykrikoides* populations and to target the timing of the Diel Studies. The methods for all sampling and analyses are described in detail in the following sections. All times given are local, UTC – 4 h, and dates are reported in DD/MM/YYYY format.

### 2.2 Diel Studies

The Diel Studies were short time series experiments designed to observe and quantify the DVM rate of *M. polykrikoides* in the field. Diel Studies were only conducted when a bloom of *M. polykrikoides* was observed to be in progress at NYCC, when *M. polykrikoides* biomass accounted for over 80% of the total phytoplankton biomass. To characterize the phytoplankton community, whole water samples were collected around 12:00 local time from 0.25 m below the surface in 50 mL conical centrifuge tubes using a Masterflex peristaltic pump and polyethylene tubing. Samples were preserved using 2% acid Lugol’s solution for later identification and enumeration of phytoplankton cells by light microscopy on a Nikon TS100 inverted microscope under brightfield illumination in the Phytoplankton Analysis Laboratory at Old Dominion University, hereafter referred to as the ODU Phyto Lab. Sample bottles were gently inverted 10 times before a Palmer Maloney Counting Cell was loaded with 0.1 mL of Lugol’s preserved whole water sample and allowed to settle for about 3–5 min. An initial scan at low magnification was conducted to ensure no bubbles were within the chamber, and that phytoplankton cells were settled properly in the viewing plane. Full taxonomic enumerations of each chamber were performed in a transect fashion while avoiding the loading channels (LeGresley and McDermott, 2010) under 300× magnification by the ODU Phyto Lab. This method was specifically chosen in order to enumerate phytoplankton from a wide range of size classes. Although a smaller magnification could be used to enumerate *M. polykrikoides*, the higher magnification aids in the enumeration of smaller cells in the densely populated and diverse samples from the estuarine environment of the Chesapeake Bay (Karlson et al., 2010). The carbon biomass for each taxa observed was calculated using the accepted Chesapeake Bay Program biovolumes and carbon conversions estimated for each species enumerated (Interstate Commission on the Potomac River Basin, 2008). At the same time, additional whole water samples (25–50 mL) were filtered onto Whatmann GF/F filters for Chl-a analysis. Chl-a concentrations were measured fluorometrically using the non-acidification method on a Turner 10-AU fluorometer after extraction in acetone (Welschmeyer, 1994) and the Chl-a data were used to calibrate the YSI fluorescence profiles (see Supplementary Figure S1). All discrete Chl-a samples were analyzed within 2 weeks of their collection.

A total of four Diel Studies were completed, three in 2016 and one in 2021, over the following dates and start/end times: 12:00 16/08/2016 to 17:00 17/08/2016 (DS1, 29 h); 12:00 22/08/2016 to 16:00 23/08/2016 (DS2, 28 h), and 16:30 09/09/2016 to 16:00 10/09/2016 (DS3, 23.5 h); 12:00 06/08/2021 to 09:00 07/08/2021 (DS4, 21 h). Each Diel Study was conducted over a period of roughly 24 h but ranged in duration from 21 to 29 h. During each Diel Study vertical profiles of temperature, salinity and Chl-a fluorescence were collected at 1–2 hourly intervals using a YSI Model 6600 sonde. Additionally, during DS1, DS2, and DS3, a subset of water samples were collected from a range of depths to characterize the phytoplankton community, albeit at a lower vertical and temporal resolution than the YSI profiles. This sampling scheme allowed us to resolve both vertical and temporal variability in water column conditions over the daily cycle.

### 2.3 Determining DVM rates

Previous studies have estimated DVM rates by tracking the relative abundance of *M. polykrikoides* with depth over the diel cycle (Park et al., 2001; Kim et al., 2010). Since the abundance of *M. polykrikoides* varies spatially as a result of biological and physical processes unrelated to DVM, by tracking the maximum relative abundance with respect to depth, we can track the DVM behavior of *M. polykrikoides* independently of variations due to spatial heterogeneity. However, unlike previous studies, here we use Chl-a as a proxy for *M. polykrikoides* biomass and track the relative concentration of Chl-a with depth, defined by Equation 1. Using Chl-a as a proxy allows for greater depth resolution and a larger number of replicate studies as it greatly reduces the labor required to analyse the data from each Diel Study. Although Chl-a is representative of the entire photoautotrophic community, Diel Studies were only undertaken during periods when *M. polykrikoides* accounted for the majority (>80%) of the total phytoplankton biomass. This allowed for the use of Chl-a as a good proxy for *M. polykrikoides* abundance. Further, we assume that the DVM behavior of *M. polykrikoides* is the same for any given water parcel in the vicinity of the study site, so that even if water parcels are moving through the fixed point in space where the sampling occurs, the timing and rate of *M. polykrikoides* DVM should be relatively invariant. Given these assumptions and constraints, we use the Chl-a concentration at any given depth relative to the total depth integrated Chl-a to track the vertical migration of *M. polykrikoides* over the course of the Diel Studies:


(1) $$Rel_{CHL}\left(z\right)=\frac{Chl-a\left(z\right)}{\int_{0}^{d}Chl-a.\;dz}$$

where the *Rel_CHL_*(z) is the proportion of total depth-integrated Chl-a at depth *z*. DVM rates, w_DVM_ (m h^−1^), were calculated from the change in z_MAX_, the depth where the maximum value of *Rel_CHL_* was identified between adjacent time points, ∆z_MAX_ (m), over the length of time between adjacent time points, ∆t (hr):


(2) $$w_{DVM}=\frac{\Delta z_{\mathrm{MAX}}}{\Delta t}$$

Chl-a data were averaged into 0.2 m bins and the frequency of sampling ranged from 1 to 2 h. The method presented here to estimate DVM rates relies on being able to identify a Chl-a maximum at some depth in the water column. When it is not possible to unequivocally identify a Chl-a maximum, such as when the water column is very well mixed, this method cannot be applied. To systematically identify periods when Chl-a was homogeneously distributed throughout the water column, we calculated the coefficient of variation of Chl-a for each of the Diel Study profiles. Here the coefficient of variation of Chl-a quantifies the relative degree of dispersion of the Chl-a values measured along each profile with respect to the mean, so when its value is low, the variability of Chl-a with depth is low. We identified profiles where the coefficient of variation of Chl-a was ≤5% and excluded those low variability profiles from our calculations of w_DVM_ in order to minimize errors or biases that might be introduced by using data where no clear Chl-a maximum could be identified.

## 3 Results

### 3.1 Environmental conditions during Diel Studies

**Figure 2:**
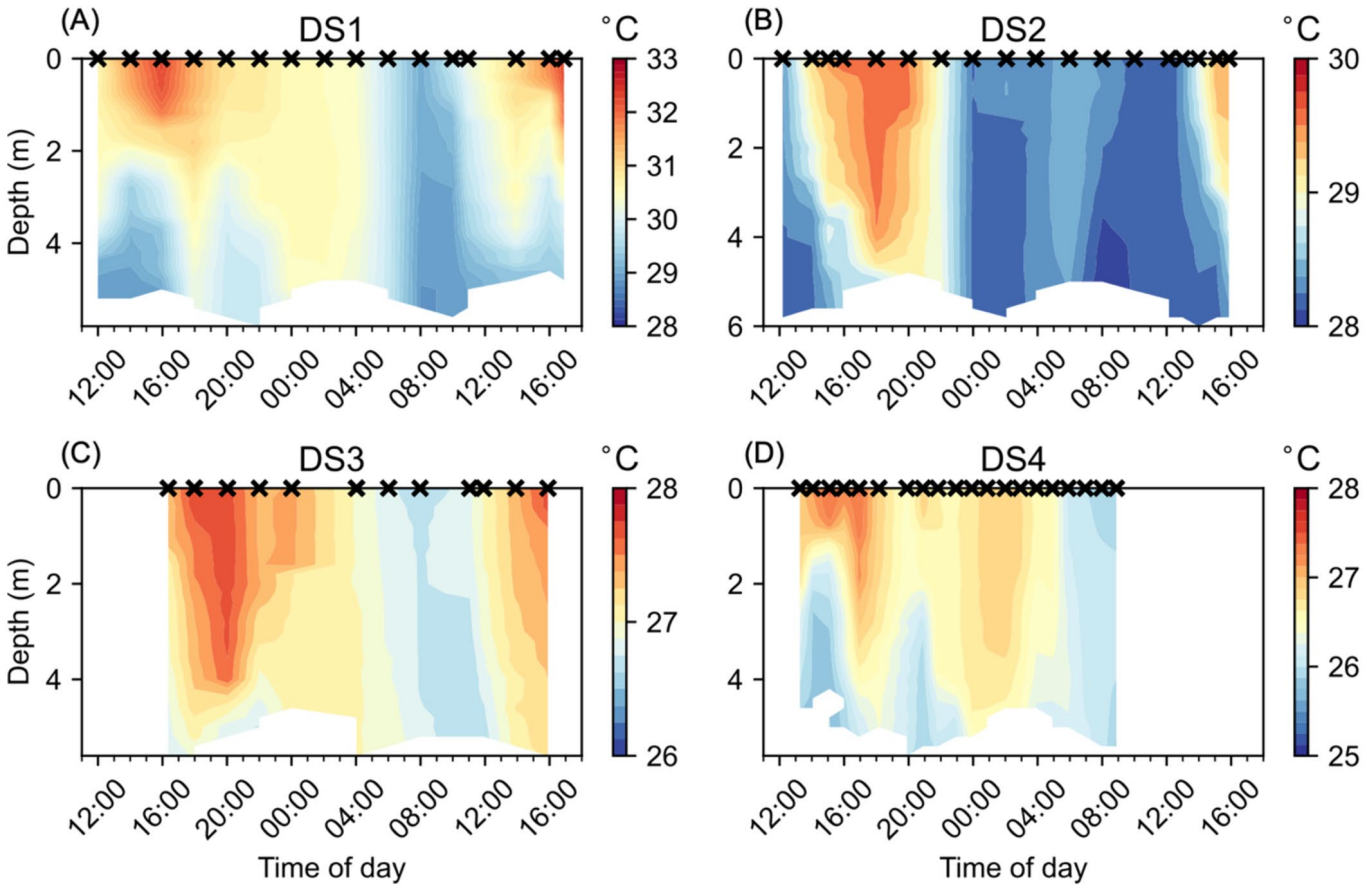
Water column temperature (°C) at NYCC during the Diel Studies, each shown on a separate panel: **(A)** DS1, **(B)** DS2, **(C)** DS3 and **(D)** DS4. The timing of each CTD cast is indicated as a cross (x) on the upper x-axis for reference, and all times are given as local times. Note the different color scales for each individual panel.

During each of the studies, the water column temperatures were clearly influenced by the diel cycle of heating and cooling (Figure 2), with the highest surface temperatures observed after noon and with their deepest penetration into the water column occurring later in the day. Water column temperatures were coolest in the early morning, although some variability was observed, likely due to differences in the tidal cycle and wind conditions between each of the studies. Water temperatures were most elevated during DS1 (Figure 2A), with water temperatures in the top 1 m exceeding 32°C in the late afternoon (16:00–17:00) on the first and second days of the study and reaching a maximum observed temperature of 32.3°C at 16:00 on 17/08/2016. The lowest water temperatures during DS1 were around 28.9°C and only found below 4.4 m depth on the morning of 17/08/2016 (08:00–10:00). Water temperatures during DS2 (Figure 2B) followed a similar pattern to DS1, but were cooler, with a maximum temperature of 29.6°C, and warm waters (>29.5°C) extending as deep as 3.6 m depth on 22/08/2016 (18:00). The minimum temperature observed during DS2 was 28.0°C and only seen below 4 m depth on the morning of 23/08/2016 (08:00). DS3 (Figure 2C) and DS4 (Figure 2D) both had much cooler water temperatures overall, with their maximum observed temperatures of 27.7°C and 27.4°C, respectively, both cooler than the lowest temperatures observed during DS1 and DS2. The lowest temperatures observed during DS3 and DS4, again were similar, at 26.6°C and 25.7°C, respectively. During each of the studies, we observed a period of relative water column temperature homogeneity, generally during the morning, coinciding with the lowest temperatures. This period was considerably longer during DS2 (Figure 2B) compared to the other studies, with relatively lower and more consistent temperatures with depth from 00:00 to 12:00. The widest range of temperatures was observed during DS1 at 3.4°C, and the lowest range of temperatures was observed during DS3 at 1.1°C. DS2 and DS4, despite the difference in the overall temperatures during the studies, had a similar range at 1.6°C and 1.7°C, respectively.

**Figure 3:**
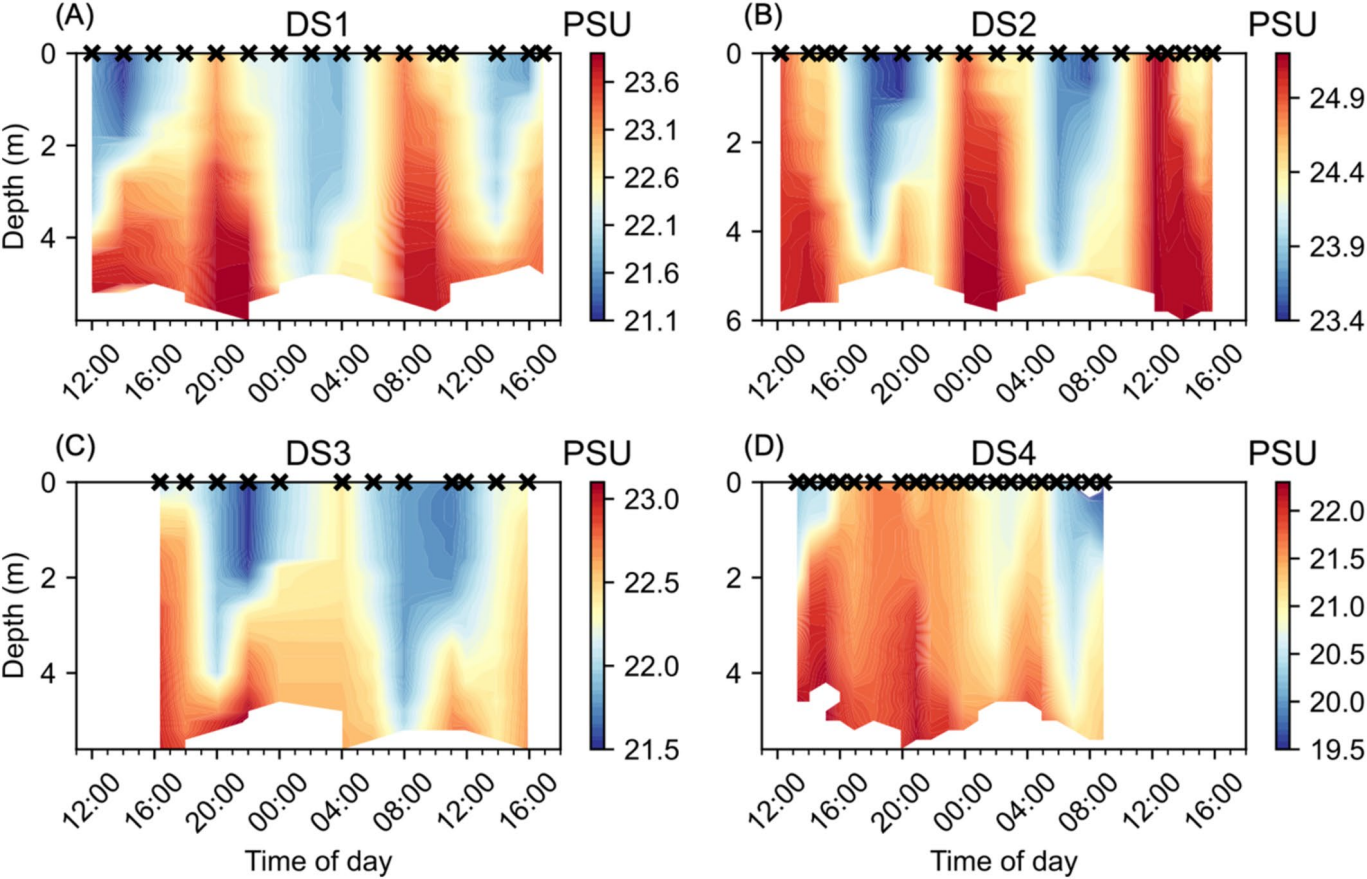
Water column salinity (PSU) at NYCC during the Diel Studies, each shown on a separate panel: **(A)** DS1, **(B)** DS2, **(C)** DS3 and **(D)** DS4. The timing of each CTD cast is indicated as a cross (x) on the upper x-axis for reference, and all times are given as local times. Note the different color scales for each individual panel.

The Lafayette River is tidal, resulting in regular sub-daily variability in salinity at NYCC during the Diel Studies (Figure 3). We observed alternating periods of high/low salinity associated with high/low tides. The relatively fresher waters were concentrated near the surface, with higher salinity waters extending through the full depth of the water column, and sometimes persisting in the deeper waters during periods of low tide, for example during the first half of both DS3 and DS4 (Figures 3C,D). We observed very similar patterns in salinity during DS1 and DS3 with salinity maxima of 23.9 PSU and 23.1 PSU, respectively, and salinity minima of 21.1 PSU and 21.5 PSU, respectively. DS2 had the highest salinities (Figure 3B), with a maximum of 25.2 PSU observed primarily lower in the water column, but extending to the surface at 12:00 on 23/08/2016, the second day of DS2. By contrast, we observed the lowest salinities during DS4 (Figure 3D), with a salinity maximum of 22.3 PSU observed below 4 m depth at 15:00 on 6/08/2021, the first day of the study. The salinity minimum during DS4 was 14.9 PSU at the surface at 08:00 on 7/08/2021, the second day of the study, this was likely a shallow lens caused by a surface input of fresh water. Apart from this extreme low salinity feature, low salinities <20 PSU were observed in the top 1 m of the water column below the fresh lens and during the subsequent profile at 09:00. The widest range of salinity was observed during DS4 at 8.7 PSU. DS1 had an intermediate range of salinity at 2.8 PSU. DS2 and DS3 had a relatively lower range of salinity at 1.8 PSU and 1.6 PSU, respectively.

### 3.2 *Margalefidinium polykrikoides* abundance during the Diel Studies

The Diel Studies were timed to coincide with periods during which *M. polykrikoides* blooms were present in the water column at NYCC. Daily abundances of phytoplankton were enumerated as part of the routine sampling at NYCC, and from that data, the proportion of the total phytoplankton biomass, as a function of carbon, represented by *M. polykrikoides* was estimated (Table 1). In most cases the abundance of *M. polykrikoides* was near or above 1,000 cells mL^−1^, and *M. polykrikoides* accounted for over 80% of the total phytoplankton biomass, except for the second day of DS2 (23/08/2016), when *M. polykrikoides* abundance was only 530 cells mL^−1^, and it accounted for 73.2% of total phytoplankton biomass in the surface. However, on the first day of DS2 (22/08/2016) when the study was initiated, *M. polykrikoides* abundance was above 10,000 cells mL^−1^ and it accounted for over 94% of the total phytoplankton biomass. Similarly, on the first day of DS3 (9/09/2016 at 16:00), *M. polykrikoides* abundance was only 890 cells mL^−1^, but it accounted for 84.8% of the total phytoplankton biomass, well above the 80% cutoff. For DS4, we report *M. polykrikoides* abundance data collected 4 days prior to, and 2 days after, the Diel Study was completed, and in both cases *M. polykrikoides* accounted for >94% of the total phytoplankton biomass. Based on the *M. polykrikoides* abundances and percentage of total biomass prior to and after DS4 as well as spot checks of the community composition undertaken with a PlanktoScope imaging microscope in the field, we are confident that *M. polykrikoides* was dominant during DS4. Unfortunately, we did not fully analyze the PlanktoScope image data, and so cannot provide abundance data from the spot checks.

**Table 1:** *M. polykrikoides* abundance and percentage of the total phytoplankton biomass from samples taken at 12:00 (local time) and 0.25 m depth at NYCC during the each of the Diel Studies.

Diel Study	Date	*M. polykrikoides* abundance (cells mL^−1^)	*M. polykrikoides* % of total phytoplankton biomass
DS1	16/8/2016	2,380	84.7
	17/8/2016	2,180	84.3
DS2	22/8/2016	10,950	94.5
	23/8/2016	530	73.2
DS3*	9/9/2016	890	84.8
	10/9/2016	3,320	93.1
DS4^+^	2/8/2021	930	94.5
	9/8/2021	1,130	94.3

* for DS3, the sample on 9/9/2016 was taken at 16:00 (local time) and not 12:00 as for the other samples; ^+^ for DS4, which was undertaken from 6/8/2021 to 7/8/2021, the reference phytoplankton samples were obtained 4 days prior to, and 2 days after, the diel study was completed.

### 3.3 Variability in Chl-a and Rel_CHL_ distributions

**Figure 4:**
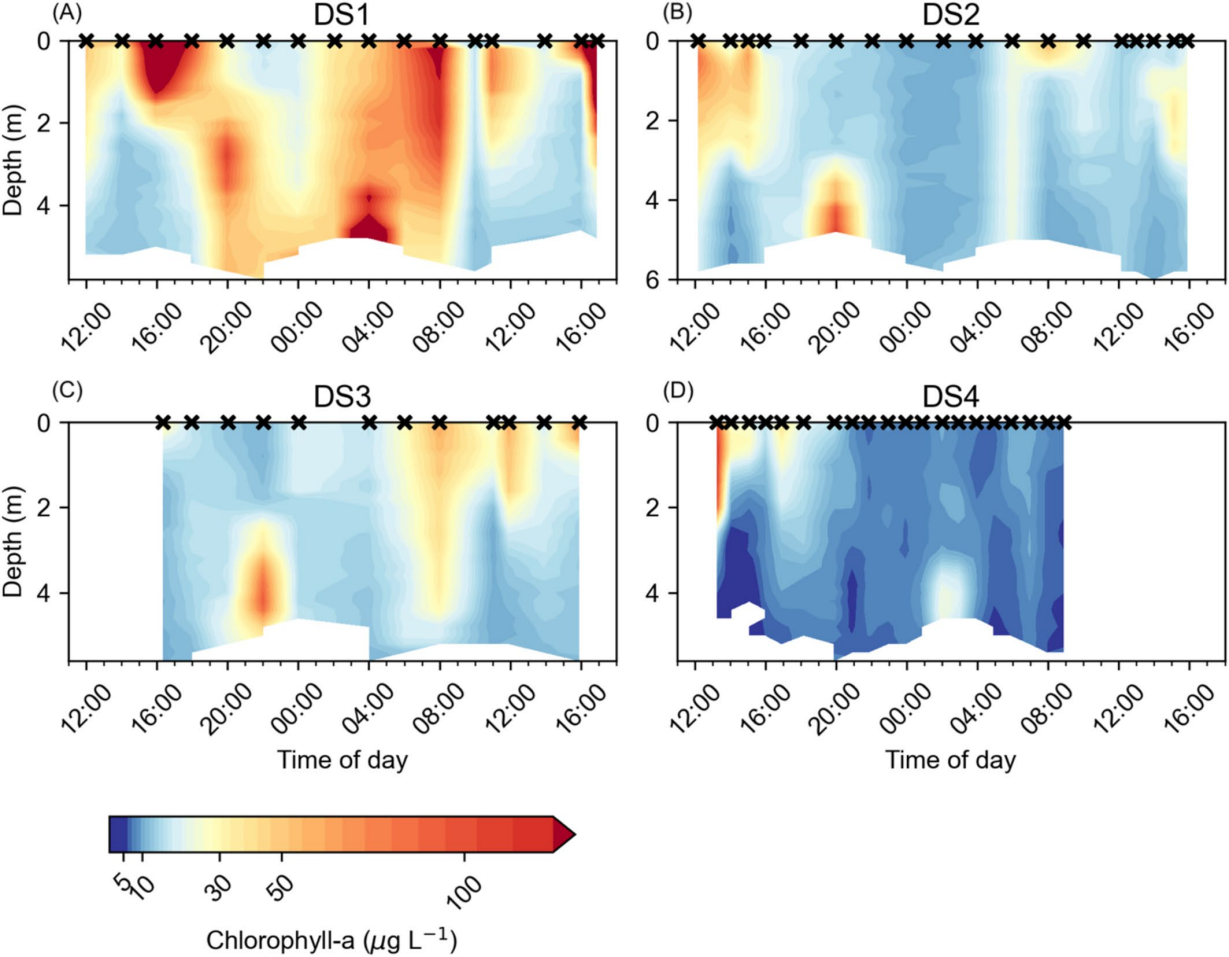
Water column chlorophyll *a* concentrations (μg L^−1^) at NYCC during the Diel Studies, each shown on a separate panel: **(A)** DS1, **(B)** DS2, **(C)** DS3 and **(D)** DS4. The timing of each CTD cast is indicated as a cross (x) on the upper x-axis for reference, and all times are given as local times. Note the logarithmic color scale.

**Figure 5:**
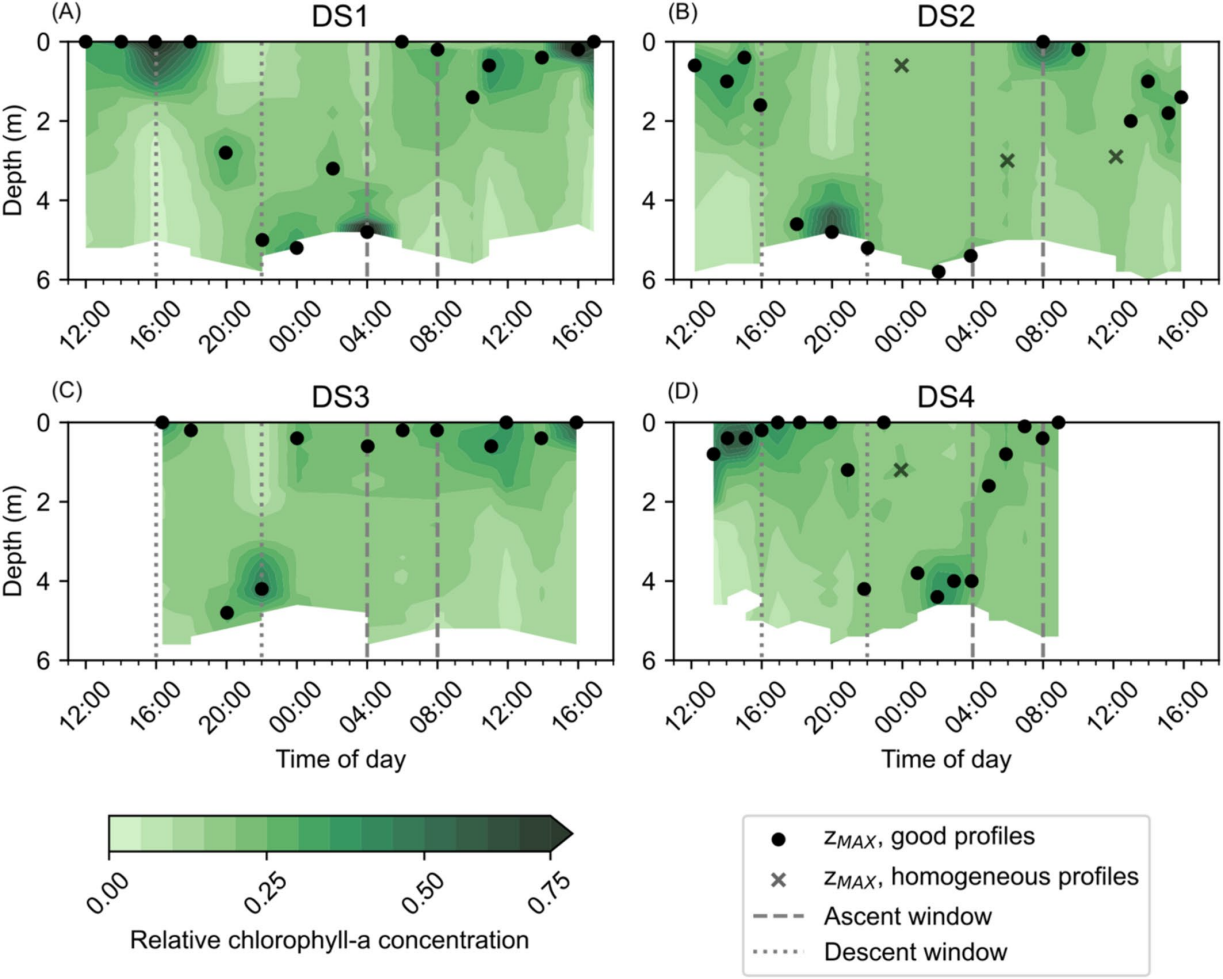
Relative Chl-a (Rel_CHL_) concentrations observed at NYCC during the Diel Studies, each shown on a separate panel: **(A)** DS1, **(B)** DS2, **(C)** DS3 and **(D)** DS4. The ascent and descent windows are indicated as gray dashed and dotted lines, respectively. The depth at which the maximum relative chlorophyll concentration is observed (z_MAX_) is shown for each profile as a filled black circle, or as a black cross in cases when the coefficient of variation of the profile was <5%, indicating a relatively homogeneous chlorophyll distribution with depth. The z_MAX_ associated with homogeneous profiles are not used in the calculation of maximum or mean DVM rates. All times are given as local times.

Temporal changes in the vertical distribution of both Chl-a (Figure 4) and Rel_CHL_ (Figure 5) were observed in each of the Diel Studies. Broadly, we observed the highest Chl-a concentrations in the surface waters during the daytime, with the pattern reversing overnight when the highest Chl-a concentrations tended to be found near the bottom (Figure 4). Across all the Diel Studies, the highest Chl-a concentrations were observed during DS1, when they reached 455.4 μg L^−1^ in surface waters around 17:00 on the first day of the study. This was an extreme high concentration, but Chl-a concentrations >200 μg L^−1^ were found to be associated with the near surface and near bottom Chl-a maxima throughout DS1. The minimum observed Chl-a concentration during DS1 was 12.0 μg L^−1^ in near bottom waters around 10:00 on the second day of the study (Figure 4A). Similar Chl-a maxima and minima were observed during the other three Diel Studies, with minima of 9.0 μg L^−1^, 9.7 μg L^−1^ and 3.7 μg L^−1^ and maxima of 108.9 μg L^−1^, 92.2 μg L^−1^ and 116.5 μg L^−1^ for DS2, DS3, and DS4, respectively (Figures 4B–D). As with DS1, the maximum Chl-a values for each study were associated with daytime near surface or nighttime near bottom maxima.

Diel patterns in the vertical distribution of Chl-a were more easily observed in the temporal evolution of Rel_CHL_, and the variation in z_MAX_ during the Diel Studies (Figure 5). In most cases, high Rel_CHL_ values and z_MAX_ were found near the bottom at night and in the early morning hours, in the surface and near surface waters between early morning and early afternoon, and in mid-depth waters and near the bottom in the late afternoon and evening. This variation in the depth distribution of Rel_CHL_ and z_MAX_ over the daily cycle reflected *M. polykrikoides’* daily vertical migration since *M. polykrikoides* vastly dominated the phytoplankton community when these studies were undertaken (Table 1). In most cases, we observed the onset of high Rel_CHL_ values in the surface waters (<2 m) as early as 09:00, persisting in the top 1-2 m of the water column until at least 16:00. Between 16:00 and 20:00, the highest values of Rel_CHL_ were observed at intermediate depths between the surface and the bottom, with high Rel_CHL_ values found mostly at the bottom from around 20:00 to 04:00 the following day. Based on these consistent patterns in the depth distribution of Rel_CHL_ and z_MAX_ over the daily cycle, we broadly defined *M. polykrikoides’* ascent window as 04:00 to 09:00, and their descent window as 16:00 to 20:00. Although this diel pattern was relatively consistent across all four Diel Studies, we particularly highlight the absence of a clear ascent pattern in Rel_CHL_ during DS3 (Figure 5C), where z_MAX_ remains confined to the top 1 m of the water column from approximately 00:00 to 16:00 on 10/09/2016 when the study ended.

**Figure 6:**
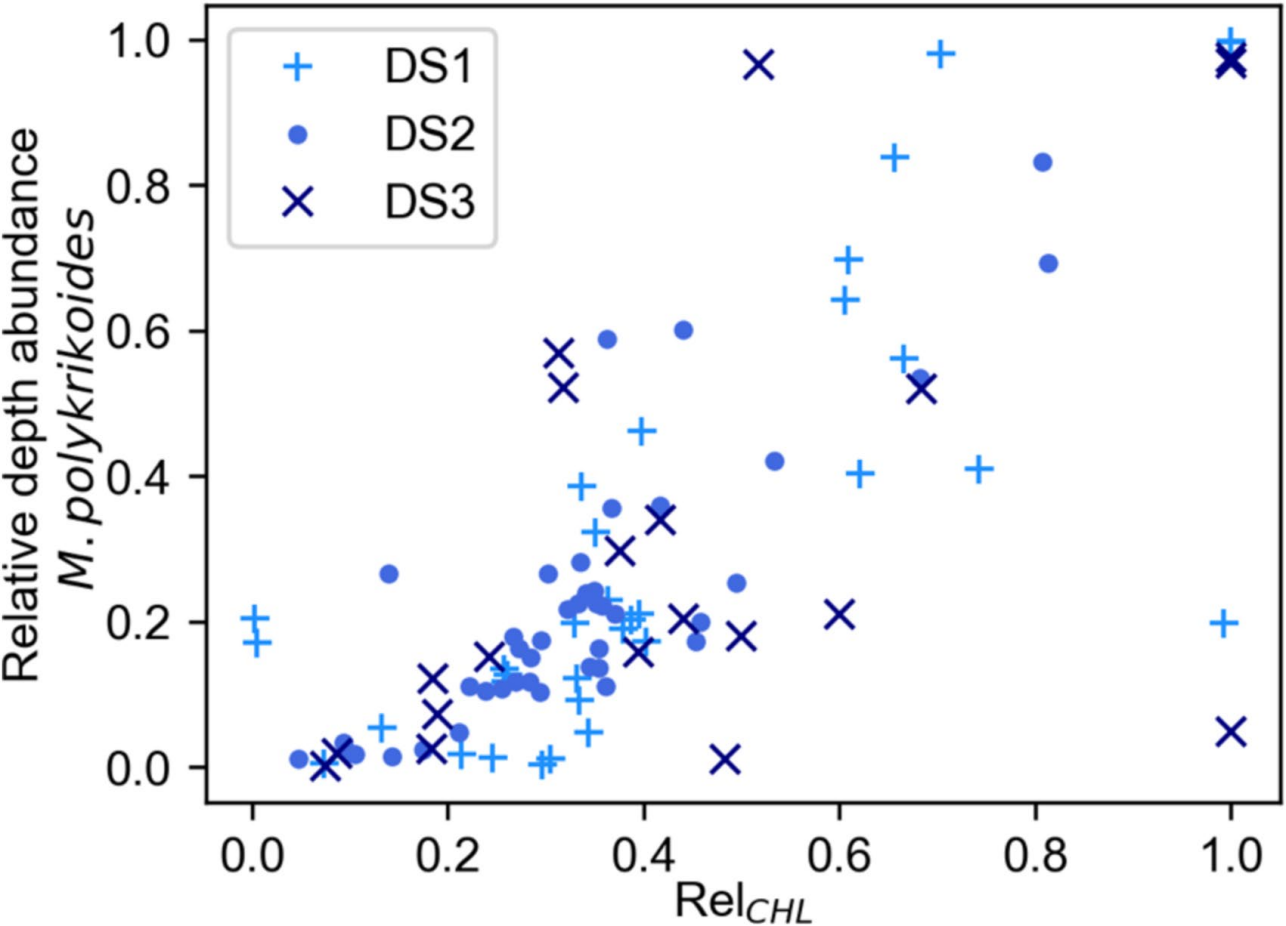
Relationship between Rel_CHL_, the Chl-a concentration relative to depth integrated Chl-a, and the relative abundance of *M. polykrikoides* with respect to depth for each profile. The data used for this plot were collected only during DS1, DS2, and DS3 from a subset of depths and time points during the Diel Studies. We found a significant (*p* < 0.01) positive Spearman Rank correlation (*r* = 0.74) between these two variables.

We examined the samples taken during DS1, DS2 and DS3 to determine whether there was a relationship between Chl-a concentration and either the abundance of *M. polykrikoides* (Supplementary Figure S1A) or its percentage of the total biomass (Supplementary Figure S1B) and did not find any strong correlation between Chl-a concentration and either of these metrics of *M. polykrikoides*. However, when we examined the relationship between Rel_CHL_ and the relative depth abundance of *M. polykrikoides* (Figure 6) we found a strong positive relationship reflected by a statistically significant (*p* < 0.01) Spearman rank correlation of r = 0.74. This strongly supports the use of Rel_CHL_ as a proxy for the vertical position of *M. polykrikoides* during bloom conditions.

### 3.4 Estimated *Margalefidinium polykrikoides* DVM rates

*M. polykrikoides* DVM rates, w_DVM_, for each of the Diel Studies were estimated based on the temporal evolution of z_MAX_, the depth of the highest Rel_CHL_, over the duration of each of the studies. Using Equation 2 we calculated a mean and maximum w_DVM_ for each ascent/descent window in each Diel Study (Table 2). The mean w_DVM_ was determined by averaging all values of w_DVM_ between the time point where *M. polykrikoides* was last observed at the surface/bottom, and the next time point where *M. polykrikoides* was observed at the bottom/surface. The maximum w_DVM_ was simply the highest observed ascent/descent rate between adjacent profiles during any given Diel Study, although in cases like DS2 when the ascent was only resolved by two sequential profiles, we did not report a maximum w_DVM_. The mean w_DVM_ for ascents ranged from 1.2 m h^−1^ for DS1 to 1.3 m h^−1^ for DS2 and DS4 (no ascent was observed in DS3), and for descents ranged from 1.2 m h^−1^ for DS1 and DS3, to 2.2 m h^−1^ for DS4. The maximum w_DVM_ for ascents ranged from 2.4 m h^−1^ for DS1 to 2.5 m h^−1^ for DS4, and for descents ranged from 1.4 m h^−1^ for DS1 and DS2 to 3.2 m h^−1^ for DS4. Given the relatively small number of replicates, 3 ascents (2 for maximum w_DVM_) and 4 descents, we cannot unequivocally say whether the mean or maximum ascents or descents were consistently faster. We also note that the fastest mean and maximum w_DVM_ values were observed during DS4, which had relatively higher resolution temporal sampling at 1.0 ± 0.2 h between casts compared to 1.9 ± 0.5 h, 1.6 ± 0.5 h and 2.1 ± 0.8 h for DS1, DS2 and DS3, respectively.

**Table 2:** *M. polykrikoides* estimated DVM ascent and descent rates (m h^−1^) for each of the Diel Studies.

Diel study	Ascent rate (m h^−1^)		Descent rate (m h^−1^)	
	Maximum	Mean	Maximum	Mean
DS1	2.4	1.2	1.4	1.2
DS2	*	1.3	1.4	1.4
DS3	Ascent not observed		2.2	1.2
DS4	2.5	1.3	3.2	2.2

In each case we report the maximum observed DVM rate and the mean DVM rate over the ascent/descent window. Where the mean DVM estimates are only based on 2 data points, as in the ascent window of DS2, we do not report a maximum DVM rate (indicated by an asterisk).

### 3.5 Presence of other vertically migrating dinoflagellates

The method presented here relies on *M. polykrikoides* being the dominant phytoplankton species present in the water column. Other vertically migrating dinoflagellate species such as *Akashiwo sanguinea* and *Gymnodinium* sp. also bloom in the Chesapeake Bay during the summer months, however they typically do not bloom at the same time as *M. polykrikoides* (Mulholland et al., 2018). An examination of the surface and water column phytoplankton community composition data collected before, after and during the Diel Studies (provided in Section S1 of Supplementary material) confirm that although other vertically migrating dinoflagellate species were present during the Diel Studies, they generally accounted for a very small proportion (<20%) of the total phytoplankton biomass, were at much lower abundances than *M. polykrikoides* and where depth-resolved data were available, were offset in time and depth from the bulk of the *M. polykrikoides* depth-resolved biomass.

## 4 Discussion

This study provides the first field estimates of *M. polykrikoides* DVM rates in the Chesapeake Bay, and our results from four individual field studies conducted during different months and years suggest that the DVM behavior of *M. polykrikoides* during blooms does not vary considerably. We found little variation in mean DVM rates (Table 2), and although there was more variability in the maximum observed DVM rates, this may be due in part to the temporal resolution of the observations as some profiles, where no distinct Chl-a maximum could be identified, were excluded from our calculations. When the length of time between profiles is reduced, it is possible to resolve increasingly faster rates. The bulk of previous studies that have quantified and described *M. polykrikoides* DVM rates and behavior in the field were undertaken in Korean coastal waters (Park et al., 2001; Kim et al., 2010; Lim et al., 2022), and we summarize their findings, along with our own results, in Table 3. As in the previous studies, our range of mean ascent rates are relatively constrained to a tight range of values, and overall, our estimated mean ascent rates are lower than those from previous studies. We note that the water depth at our estuarine study site, 6 m, is considerably shallower than the Korean coastal sites which ranged from 12 to 20 m depth. Given the shorter distance to travel between the bottom and the surface, it is possible that the *M. polykrikoides* strain found in the Chesapeake Bay may swim more slowly, on average, to reach the surface. If this is the case, then it may be that *M. polykrikoides’* DVM is timed to bring them into surface waters during a specific window of time, which can be reached by swimming more slowly over a shallower water column. Since *M. polykrikoides’* initial departure from the bottom waters consistently occurred before sunrise, generally at around 04:00, our results suggest that their ascent is not triggered by light, agreeing with previous work (Lim et al., 2022). However, light levels or sun angle may play a role in regulating ascent rates once they are moving through the water column, since we did find that the rate of ascent varied during the ascent window. This is particularly evident during DS4, when the rate of ascent appeared to slow after an initially fast ascent from 04:00 to 05:00 (Figure 5D). Unfortunately, we did not measure surface or ambient light levels during the Diel Studies, but we have obtained data on the sunrise, sunset and solar noon times along with the day lengths, which are given in Supplementary Table S1. Although the overall day length varied by 1 h and 13 min across the studies, the solar noon times were relatively invariant with a range of only 9 min between the earliest and latest. DS4 had the earliest sunrise (06:14) and the longest day length (13 h and 50 min) of the Diel Studies, and so it is possible that during DS4, the resident *M. polykrikoides’* population slowed their morning ascent rate to account for the longer period between sunrise and solar noon compared to the other Diel Studies. Sunrise times varied by 28 min, and sunset times varied by 46 min across the Diel Studies and so sampling at higher temporal frequency would be needed to identify their impact on the timing of *M. polykrikoides’* DVM. The range of previously reported descent rates was very wide, ranging from 0.47 m h^−1^ to 4 m h^−1^, and our descent rates all fall within that range. Previous work has suggested that *M. polykrikoides’* descent rates are higher than their ascent rates thanks to the effect of gravity (Park et al., 2001), however our results do not unequivocally support that hypothesis. Our highest reported maximum DVM rate (3.2 m h^−1^) was recorded during a descent, however the range of mean and maximum descent rates observed during our study (Table 2) are not significantly different from the ascent rates. We suggest that as with the ascents, the depth of the water column is likely playing a role in modulating *M. polykrikoides’* swimming speed, driving the similar ascent and descent rates observed at NYCC, as well as explaining the generally lower values than observed in deeper coastal settings. Although this study represents a larger number of replicate DVM rate estimates from the field (3 ascents, 4 descents) than previous ones, this number is still too small to state with any certainty that the range of descent rates are significantly higher than the ascent rates.

**Table 3:** Ranges of *M. polykrikoides’* mean ascent and descent rates (m h^−1^) derived from field data reported in this and previous published studies.

	Ascent rate (m h^−1^)	Descent rate (m h^−1^)	Location	Water depth (m)
This study	1.2–1.3	1.2–2.2	Chesapeake Bay, United States	6
Park et al. (2001)	3	4	Namhae Bay, East China Sea, Korea	15–20
Kim et al. (2010)	1.7–2.2	1.4–1.8	Ganggu, East Japan Sea, Korea	15
Lim et al. (2022)	1.41–1.67	0.47–2.49	Site T, Tongyeong, Korea	12–15

We report the data with the number of significant figures given in the original publication from which it was derived.

A significant anomaly in our results was the failure to detect a DVM ascent signal during DS3 (Figure 5C). Rather than observing a clear signal of high Rel_CHL_ persisting at depth from the evening through to the following early morning, our estimates of z_MAX_ jumped from the bottom to the surface between 22:00 and 00:00 and remained near the surface for the remainder of DS3. This signal is also clear in the distribution of Chl-a during DS3 (Figure 4C), with higher Chl-a concentrations found in the surface compared to depth from 00:00 onwards. It is unlikely that this is due to water column mixing since we do not see a corresponding signal in either temperature (Figure 2C) or salinity (Figure 3C), and the wind speeds were < 10 m s^−1^ (reported for the nearby Norfolk Naval Air Station, data provided by NOAA) during the study.

Water temperatures during DS1 were well above *M. polykrikoides*’ optimal thermal range of 21–26°C (Kim et al., 2004), and were higher than 30°C, a threshold for “unfavorable” thermal conditions (Lim et al., 2022). However, unlike in the field study reported in Lim et al. (2022) where *M. polykrikoides* remained up to 5 m below the surface to avoid high surface temperatures, we observed no evidence that *M. polykrikoides* modulated their DVM to avoid the hottest surface waters during DS1, which reached a high of 32.3°C (Figure 2A). During DS1, the water temperature throughout the water column was well above *M. polykrikoides’* thermal optimum, and for periods of several hours at a time was “unfavorable” based on the 30°C threshold. As a result, there was no thermal refuge available to migrate to. We propose two explanations for this apparent difference. Firstly, that the shallower waters at NYCC likely influence and constrain *M. polykrikoides’* DVM behavior, and that with no thermal refuge available, there is no benefit to moving deeper into the water column during periods when light is available for photosynthesis. Secondly, that the strain of *M. polykrikoides* found at NYCC is better adapted to the higher temperatures that are common to this estuarine system in the summer when it blooms, and so their thermal optimum and “unfavorable” threshold are both higher than those reported in the literature.

As discussed above, our estimates of *M. polykrikoides* DVM rates, derived using Chl-a fluorescence profiles rather than cell counts, are broadly comparable to those found in previous field studies based on cell counts (Park et al., 2001; Kim et al., 2010; Lim et al., 2022). However, we aim to highlight here what we see as the advantages and limitations of using Chl-a fluorescence profiles rather than cell counts for this purpose. The robustness of any estimate of DVM rates will depend on the resolution of the data across the following dimensions: length (i.e., depth), time and taxonomic resolution. The use of cell counts to estimate DVM rates provides very high taxonomic resolution, as the abundance of the species of interest can be isolated from any other constituents of the phytoplankton community also present in the water column. The trade-off, however, is likely to be lower resolution in both length and time, as collecting whole water samples for later identification imposes limitations on the number of samples that can be collected and processed. Profiles of fluorescence can provide very high resolution in depth. In this study we binned and averaged data over 0.2 m bins, thus giving an uncertainty of +/− 0.1 m on our DVM rate estimates. The vertical resolution of sampling in studies based on cell counts was in some cases variable, ranging from 1 m near the surface to 5 m at depth (Park et al., 2001; Kim et al., 2010), or constant at 6 m between samples (Lim et al., 2022). Such coarse resolution in vertical sampling results in large uncertainties in the derived DVM rates. For field sites such as ours, where the water depth is relatively shallow, more highly resolved depth measurements allow for more precise estimates of DVM rates, and better resolution of patterns in DVM behavior. The trade-off of lower taxonomic resolution when using fluorescence profiles can be mitigated, as in this study, by only undertaking studies to derive DVM rates when the migrating species of interest is significantly dominant in the community. This in turn limits the periods during which such studies can be undertaken using fluorescence and will not provide useful information on DVM behavior when a bloom is in the process of initiating or when it is in decline. However, the use of phytoplankton imaging instruments (e.g., FlowCam, Imaging Flow CytoBot, PlanktoScope) for phytoplankton identification and quantification could be used to enable an increase in both taxonomic and temporal resolution in future studies of dinoflagellate DVM, allowing for a larger number of replicate estimates of DVM rates during a broader range of conditions including bloom initiation and decline.

## 5 Conclusion

Here we have reported the first field-derived estimates of *M. polykrikoides* DVM rates for the Chesapeake Bay. We have shown that during bloom conditions, Chl-a fluorescence can be employed as a proxy for the biomass of the dominant species to investigate their vertical distribution and its evolution over the daily cycle. We envisage that by using profiles of fluorescence rather than cell counts from a limited number of depth points, a larger number of more precise DVM rate estimates can be made over the course of a bloom, particularly if a profiling mooring can be deployed in a known bloom initiation hotspot such as the Lafayette River, our study site. We found no evidence of thermotaxic influence on *M. polykrikoides’* DVM rates at our study site despite differing temperature ranges across our four Diel Studies, and temperatures during DS1 exceeding *M. polykrikoides’* ideal thermal niche.

*M. polykrikoides* annually forms large toxic blooms in the Chesapeake Bay, and efforts to monitor, predict and mitigate its harmful effects on the ecosystem rely on developing a better understanding of its behavior. It is widely believed that *M. polykrikoides’* daily migrations up and down the water column give it a competitive advantage over other phytoplankton species, allowing it to massively outcompete them and generate wide-ranging and long-lasting blooms. Existing coupled physical-biological models used for HAB modeling and prediction (Hofmann et al., 2021; Xiong et al., 2022) currently do not resolve *M. polykrikoides’* ecologically important pattern of DVM behavior. The DVM rate estimates derived as a result of this study provide invaluable ground-truth data to develop and improve model parameterizations for *M. polykrikoides’* daily migrations based on replicated field observations.

## Data Availability

The data used in this study have been deposited in the Zenodo data repository and can be freely accessed from the following link: https://doi.org/10.5281/zenodo.13986851.
