# Supplementary material for: Diel vertical migration rates of the dinoflagellate species Margalefidinium polykrikoides in a lower Chesapeake Bay tributary
Source: Front Microbiol. 2024 Nov 13;15:1378552. doi: 10.3389/fmicb.2024.1378552 (PMC11601073; doi:10.3389/fmicb.2024.1378552)
Supplement: Supplementary file 1 [file Data_Sheet_1.pdf]

Supplemental Information

S1. Presence of other vertically migrating dinoflagellates

Other vertically migrating dinoflagellate species such as *Akashiwo sanguinea* and Gymnodinium sp. are present in the Chesapeake Bay during the summer months, but typically do not bloom at the same time as *M. polykrikoides* (Mulholland et al., 2018). Additionally, based on the phytoplankton community composition data collected before, during and after the Diel Studies, we found the following:

DS1: Gymnodinium had a maximum abundance of 630 cells/mL and generally accounted for less than 10% of the total phytoplankton biomass. In a small number of samples it accounted for a larger proportion of the community, but this was limited to deeper samples (< 3.5 m) and between 1200 and 1600 (local time) when *M. polykrikoides* was predominantly shallower in the water column. *A. sanguinea* was also present during DS1, but had a maximum observed abundance of 30 cells/mL and never accounted for more than 12% of the total phytoplankton biomass.

DS2: Gymnodinium had a maximum abundance of 410 cells/mL and generally accounted for less than 20% of the total phytoplankton biomass. In a small number of samples it accounted for up to 43% of the phytoplankton biomass, but generally offset in depth and time from where *M. polykrikoides* dominated the community. *A. sanguinea* was present in a small number of samples during DS2, but had a maximum observed abundance of 20 cells/mL and never accounted for more than 2% of the total phytoplankton biomass.

DS3: Gymnodinium had a maximum abundance of 450 cells/mL and generally accounted for less than 20% of the total phytoplankton biomass. In a small number of samples it accounted for up to 43% of the phytoplankton biomass, but generally offset in depth and time from where *M. polykrikoides* dominated the community. *A. sanguinea* was present in a small number of samples during DS2, but had a maximum observed abundance of 20 cells/mL and never accounted for more than 6% of the total phytoplankton biomass.

DS4: Only midday surface water samples were available for DS4. Gymnodium was present at very low abundance (20 cells mL^{-1}) in near surface waters on 2/8/2021 before DS4, but not on 9/8/2021 after DS4. *A. sanguinea* was not observed in the near surface samples before or after DS4.

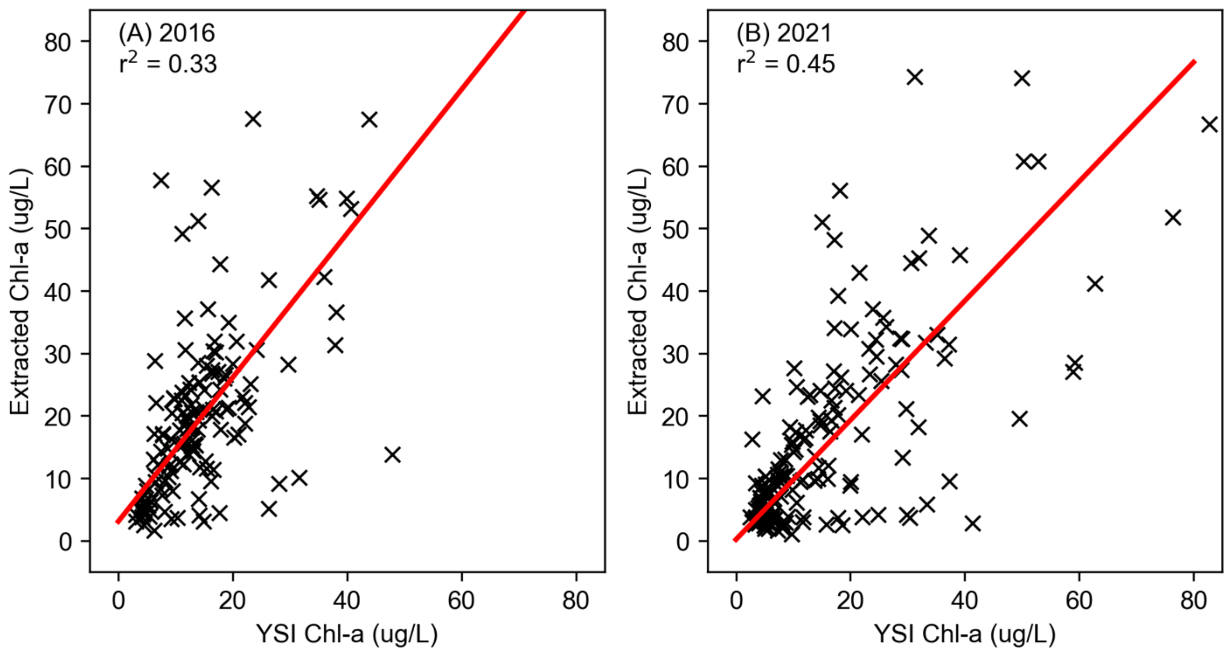

Figure S1. Chlorophyll data used to calibrate the YSI fluorometers from 2016 (A) and 2021 (B). The extracted chl-a samples are taken from a range of depths from 0.25m to 5m during the course of the summer sampling season. YSI fluorescence data is converted to chl-a concentrations using the provided manufacturer calibration and then matched up in time and depth with the extracted chl-a samples. The resulting calibration relationship (read line), based on a linear regression, is used to adjust the YSI chl-a data.

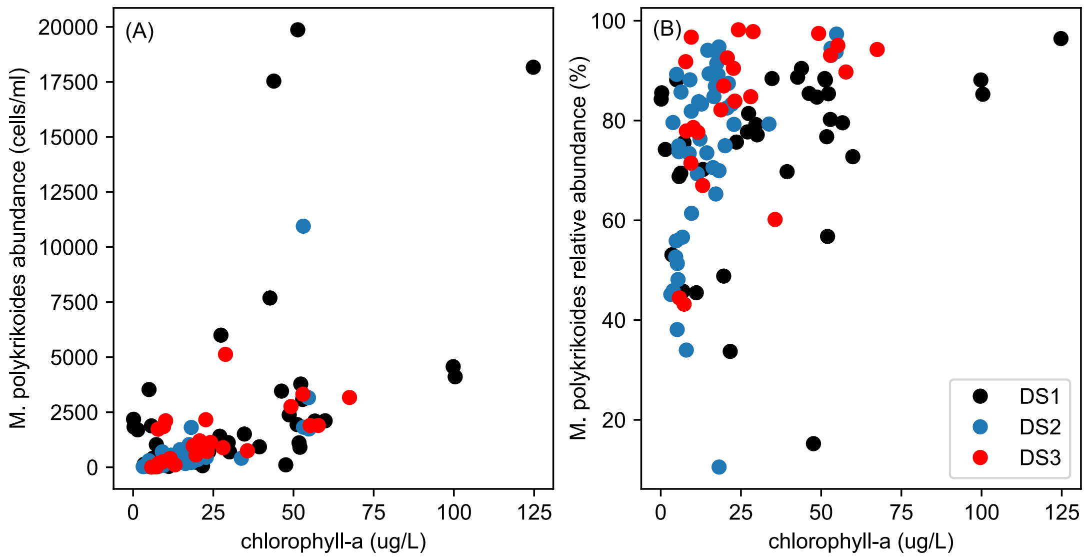

Figure S2. *M. polykrikoides* abundance (cells/ml; panel A) and relative abundance (%, panel B) plotted against Chl-a concentration (µg/L) from samples taken during DS1 (black), DS2 (blue) and DS3 (red). The samples were taken from a range of depths at 2-4 hour intervals during the diel studies.

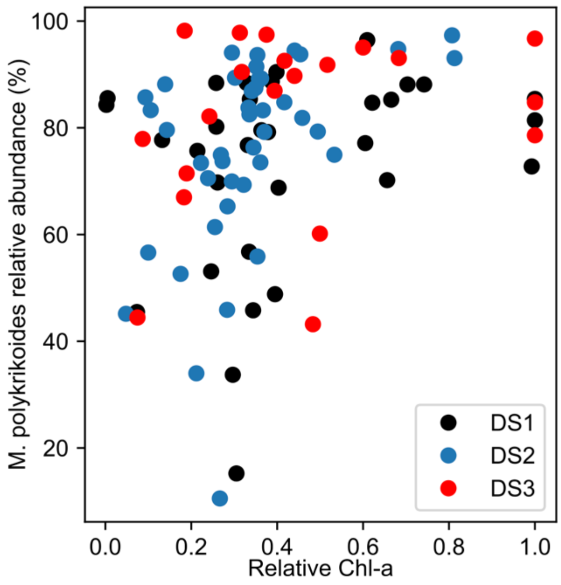

Figure S3. *M. polykrikoides* relative abundance (% total phytoplankton biomass) and plotted against relative Chl-a concentration with depth, from samples taken during DS1 (black), DS2 (blue) and DS3 (red). The samples were taken from a range of depths at 2-4 hour intervals during the diel studies.

Table S1: Sunrise, solar noon, sunset and day lengths for each of the Diel Studies. Sunrise is given for the second calendar day of the DS, sunset is given for the first calendar day of the DS, and solar noon and day length are given as the average over both calendar days of the DS. All quantities were calculated using the online NOAA Solar Calculator (https://gml.noaa.gov/grad/solcalc/), and all times are given as local times.

Diel Study	Sunrise (day 2)	Solar noon (avg)	Sunset (day 1)	Day length (hr:mm)
DS1	06:24	13:09	19:55	13:31
DS2	06:29	13:08	19:47	13:18
DS3	06:44	13:02	19:21	12:37
DS4	06:16	13:11	20:07	13:50

The day lengths of the different DS were very similar: DS4 was the longest day at 13:50 with the earliest sunrise (06:16) and latest sunset (20:07), and DS3 was the shortest day at 12:37 with the latest sunrise (06:44) and earliest sunset (19:21). Overall, there was only 9 minutes variation in the solar noon times, but day length varied by 1:13, sunsets by 46 minutes and sunrises by 28 minutes.
